# Bioactive glass grants equivalent fusion compared to autologous iliac crest bone for ALIF: a within-patient comparative study

**DOI:** 10.1186/s40634-022-00496-6

**Published:** 2022-06-17

**Authors:** Marc Szadkowski, Sami Bahroun, Ivan Aleksic, Michiel Vande Kerckhove, Sonia Ramos-Pascual, Mo Saffarini, Vincent Fière, Henri d’Astorg

**Affiliations:** 1 Ramsay Santé, Hôpital Privé Jean Mermoz, Lyon, France; 2 ReSurg SA, Rue Saint-Jean 22, 1260 Nyon, Switzerland

**Keywords:** Bioactive glass, ALIF, Bridwell grade, Fusion, Complications

## Abstract

**Purpose:**

To determine within-patient fusion rates of chambers filled with bioactive glass versus autologous iliac crest bone on computed tomography (CT) following anterior lumbar interbody fusion (ALIF).

**Methods:**

A consecutive series of 40 patients (58 levels) that underwent single-level (L5-S1 only) or two-level (L5-S1 and L4-L5) ALIF were assessed. Indications for fusion were one or more of the following: degenerative disc disease with or without Modic changes, spondylolisthesis, and stenosis. Each intervertebral cage had a middle beam delimiting two chambers, one of which was filled with bioactive glass and the other with autologous iliac crest bone. CT scans were graded using the Bridwell classification (grade I, best; grade IV, worst). Patients were evaluated using the Oswestry Disability Index (ODI), and by rating pain in the lower back and legs on a Visual Analog Scale (pVAS); complications and reoperations were noted.

**Results:**

At 15 ± 5 months follow-up, there were no significant differences in fusion across chambers filled with bioactive glass versus chambers filled with autologous bone (*p* = 0.416). Two patients with Bridwell grade III at both chambers of the L4-L5 cages required reoperation using posterior instrumentation. Clinical assessment of the 38 remaining patients (54 levels) at 25 ± 2 months, revealed ODI of 15 ± 12, lower back pVAS of 1.4 ± 1.5 and legs pVAS of 1.9 ± 1.6.

**Conclusions:**

For ALIF at L5-S1 or L4-L5, within-patient fusion rates were equivalent for bioactive glass compared to autologous iliac crest bone; thus, bioactive glass can substitute autologous bone, avoiding increased operative time and blood loss, as well as donor site morbidity.

## Introduction

Spinal fusion is a common surgical procedure, with over 400,000 surgeries performed in the United States every year [23]. Fusion is used increasingly for the treatment of spondylolisthesis, scoliosis, disc degeneration, herniation and stenosis [12, 18]. Its main goal is to fuse two or more vertebrae by inducing bone growth between segments, though fusion is not always successful, with pseudarthrosis reported in up to 50% of cases [8]. In 2016, a meta-analysis reported that patients with successful fusion had better improvements in clinical outcomes compared to patients with pseudarthrosis [21].

Autologous iliac crest bone is the gold standard graft material used during spinal fusion [24]. Harvesting autologous iliac crest bone has been associated with increased operative time and blood loss, donor site pain and morbidity, as well as increased complication rates [14, 22, 25]. Therefore, synthetic alternatives to autologous iliac crest bone graft continue to be developed and evaluated [24], of which various formulations of bioactive glass have shown promising results, when used alone or in combination with autologous bone [8].

For the last five years, the authors have been performing anterior lumbar interbody fusion (ALIF) for a variety of indications, using intervertebral cages with one chamber filled with bioactive glass and the other chamber filled with autologous iliac crest bone, within the same patient. The aim of this study was to determine the fusion rates of chambers filled with bioactive glass versus autologous iliac crest bone, within the same patient, on computed tomography (CT) following ALIF. The hypothesis was that there would be no differences in fusion rates of chambers filled with bioactive glass compared to those filled with autologous iliac crest bone.

## Materials and methods

The authors retrospectively assessed a consecutive series of 40 patients that underwent ALIF at L5-S1 between November 2017 and April 2019, operated on by 2 surgeons (BLINDED). Twenty-two patients had single-level ALIF (L5-S1 only), whereas 18 patients had two-level ALIF (L5-S1 and L4-L5). Each of the 58 intervertebral cages (L5-S1 and L4-L5) had a middle beam delimiting two chambers, one of which was filled with bioactive glass, and the other was filled with autologous iliac crest bone. Indications for ALIF surgery were one or more of the following: degenerative disc disease with or without Modic changes, spondylolisthesis, and stenosis. Posterior fixation was used in 24 patients (60%) that either had spondylolisthesis or required posterior spinal decompression (these patients required posterior incisions, so screws were added to increase stability). None of the patients had prior spine surgery, other than foraminotomy or lumbar discectomy, nor did any patients require fusion at other levels.

Standing lateral radiographs were performed to measure disc height and magnetic resonance images (MRI) were acquired to assess disc degeneration, considering modified Pfirmann grade ≥ 4 and/or Modic changes to indicate degenerative disc disease (DDD). Patients were managed conservatively for at least 1 year, and if pain persisted, surgical intervention was discussed with a physiatrist. All patients provided written informed consent to use their data and images for research and publication purposes. The study was approved in advance by ‘GCS Ramsay Santé pour l’Enseignement et la Recherche’ (IRB#: COS-RGDS-2021-05-004-SZADKOWSKI-M).

### Surgical technique

The same pre-operative protocol was used by both surgeons. Surgery was performed under general anesthesia with the patient in supine position, using a left retroperitoneal approach and implanting an ALIF intervertebral cage. Each ALIF cage had a middle beam delimiting two chambers. Grafting was performed as follows, systematically by the two surgeons: one chamber was filled with bioactive glass putty only (Glassbone®, Noraker, Lyon, France), and the other chamber was filled with autologous bone only (obtained from the patient’s iliac crest). The bioactive glass putty had a composition of 45% SiO_2_, 24.5% Na_2_O, 24.5% CaO, and 6% P_2_O_5_. The implants used at L5-S1 included both Roi A cages (*n* = 7; Zimmer Biomet, Warsaw, IN, USA) and Idys ALIF cages (*n* = 33; Clariance, Beaurains, France), while at L4-L5 they included both Roi A cages (*n* = 12; Zimmer Biomet, Warsaw, IN, USA) and Synfix cages (*n* = 6; DePuy Synthes, Raynham, MA, USA).

### Clinical and radiographic assessment

CT scans were routinely performed at 12 months, and two experienced readers (MS, SB) assessed fusion using the Bridwell classification (grades I-IV): grade I indicated fusion with remodeling and trabeculae present; grade II indicated an intact graft, not fully remodeled and incorporated, but without lucency present; grade III indicated an intact graft, with potential lucency present at the top and bottom of the graft; and grade IV indicated absence of fusion with collapse/resorption of the graft [6]. Only patients with persistent back pain after surgery or worsening clinical scores had further radiographic follow-up, to not re-expose all patients unnecessarily to additional radiation. Clinical assessment was performed preoperatively and at 3, 6, 12, and 24 months using the Oswestry Disability Index (ODI; 0–100%) and Short Form 12 (SF-12) questionnaires, and rating pain in the lower back and legs on a Visual Analog Scale (pVAS; 0–10). Only the latest follow-up of 24 months is shown in the present study. All complications, reoperations and revisions were noted.

### Statistical analysis

Descriptive statistics were used to summarize the data. Comparisons of fusion rates between autologous bone and bioactive glass were performed using Chi-squared tests. Agreement on fusion rates between the two readers were calculated using Gwet’s AC [9], and were found to be good to excellent (Gwet’s AC > 0.691; *p* < 0.001) [7]. Patients were stratified to determine whether the addition of posterior instrumentation affected clinical outcomes. Statistical analyses were conducted using R version 3.6.1 (R Foundation for Statistical Computing). *P*-values < 0.05 were considered statistically significant.

## Results

The initial cohort comprised 40 patients, 26 females and 14 males, with an age at index surgery of 49 ± 10 years and a BMI of 26 ± 3 kg/m^2^ (Table 1). Fifteen patients (38%) were smokers, all of whom confirmed to have stopped smoking at least 8 weeks before surgery. There were two early postoperative complications (5%); one hematoma and one radiculopathy, neither of which required reoperation.

**Table 1:** Patient demographics and surgical data

	**Initial cohort (*n* = 40)**		**No posterior instrumentation (*n* = 16)**		**Posterior instrumentation (*n* = 24)**	
	mean ± *SD*	(range)	mean ± *SD*	(range)	mean ± *SD*	(range)
	n (%)		n (%)		n (%)	
**Age (years)**	48.7 ± 9.8	(29 – 65)	47.3 ± 8.9	(34 – 65)	49.7 ± 10.4	(29 – 65)
**BMI (kg/m**^**2**^**)**	25.8 ± 3.5	(18 – 39)	26.0 ± 4.6	(20 – 39)	25.6 ± 2.7	(18 – 30)
**Female**	26 (65%)		11 (69%)		15 (63%)	
**Smokers**	15 (38%)		6 (38%)		9 (38%)	
**Diabetes**	1 (3%)		0 (0%)		1 (4%)	
Indications at L5-S1*						
DDD	26 (65%)		15 (94%)		11 (46%)	
Modic changes	7 (18%)		4 (25%)		3 (13%)	
Spondylolisthesis	11 (28%)		0 (0%)		11 (46%)	
Stenosis	23 (58%)		11 (69%)		12 (50%)	
**Levels fused**						
L5-S1	22 (55%)		11 (69%)		11 (46%)	
Both	18 (45%)		5 (31%)		13 (54%)	
**Type of cage at L4-L5**						
Roi A (Zimmer Biomet)	12 (30%)		0 (0%)		12 (50%)	
Synfix (DePuy Synthes)	6 (15%)		5 (31%)		1 (4%)	
None	22 (55%)		11 (69%)		11 (46%)	
**Type of cage at L5-S1**						
Roi A (Zimmer Biomet)	7 (18%)		1 (6%)		6 (25%)	
Idys ALIF (Clariance)	33 (83%)		15 (94%)		18 (75%)	

*Abbreviations: BMI* Body Mass Index, *DDD* Degenerative Disc Disease, *SD* Standard Deviation, *n* number of patients

^*^ Subgroups are not mutually exclusive

**Fig. 1:**
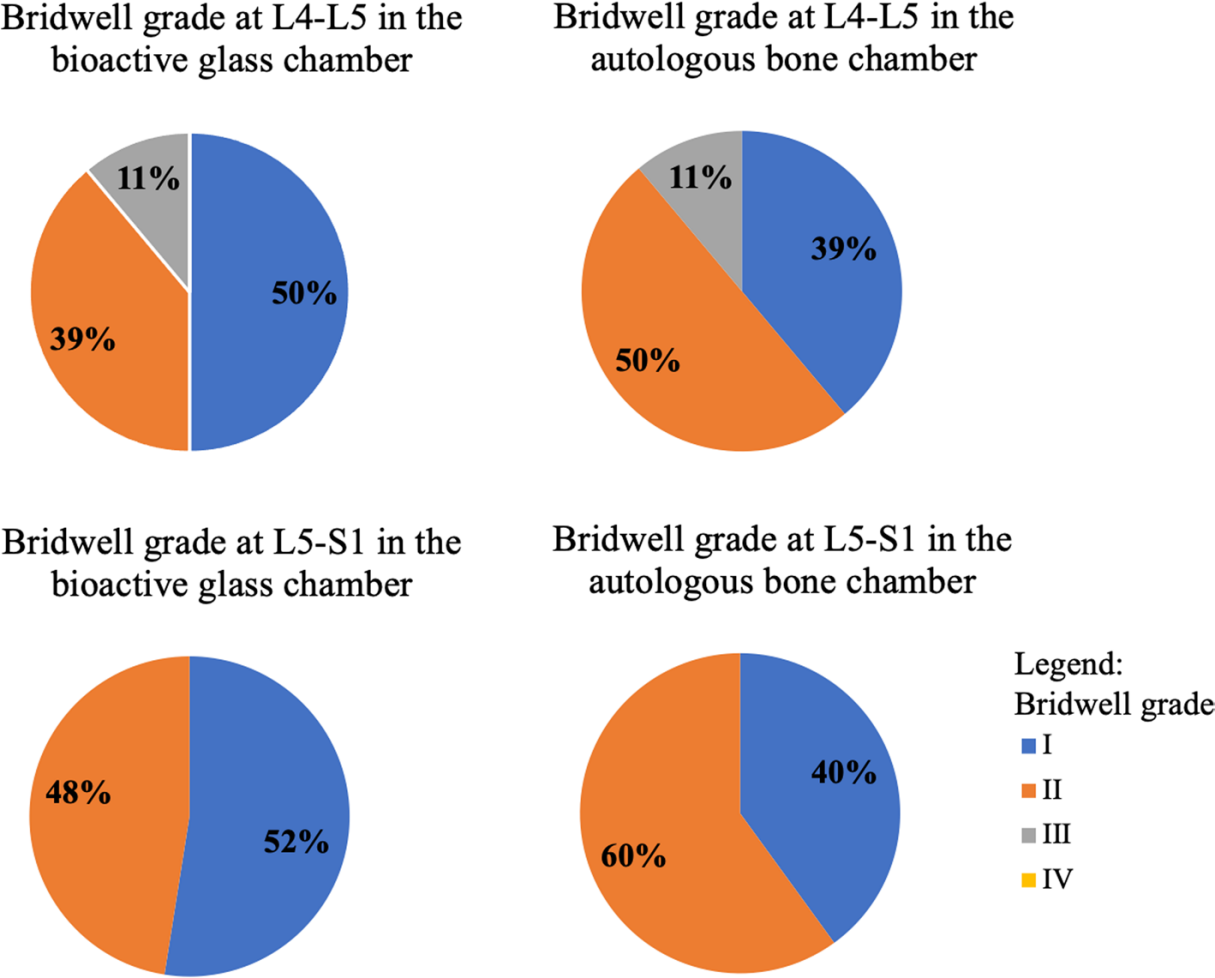
Bar chart presenting the Bridwell grades in the chambers filled with bioactive glass and autologous bone, at L4-L5 and L5-S1

**Fig. 2:**
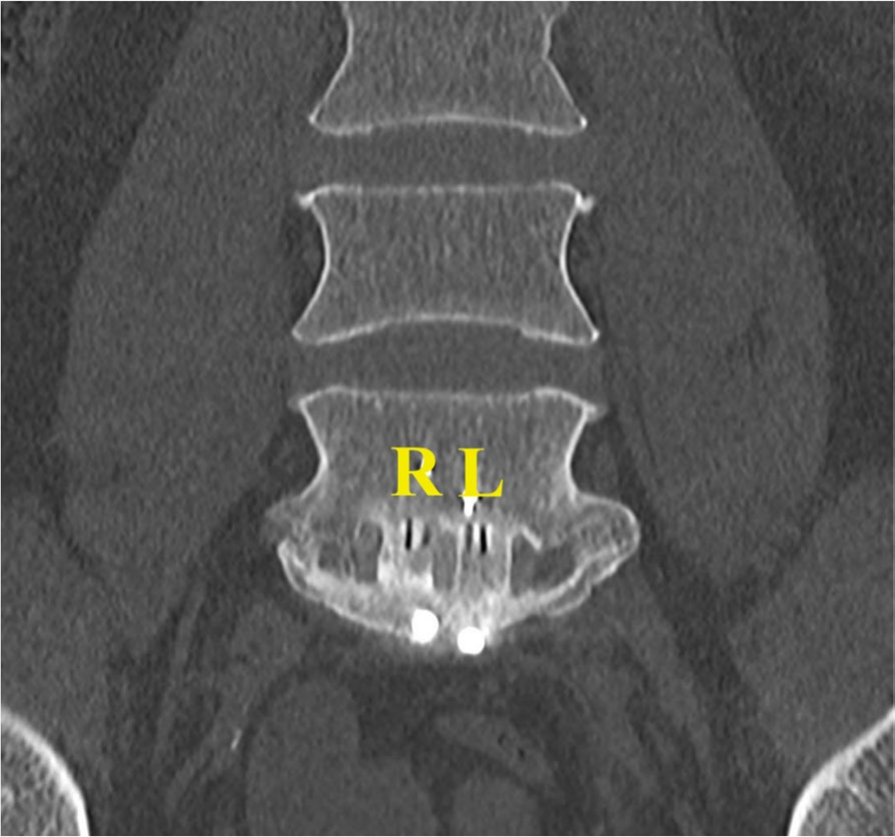
Frontal CT scan of a patient with fusion of Bridwell grade I1 in both the chamber filled with bioactive glass (R) and the chamber filled with autologous bone (L)

At a mean follow-up of 15 ± 5 months (range, 10–24), CT scans of the 40 patients (58 levels) indicated no significant differences in fusion across chambers filled with bioactive glass versus chambers filled with autologous bone (*p* = 0.416), with Bridwell grade I at 30 levels (52%) in chambers with bioactive glass versus 23 levels (40%) in chambers with autologous bone, Bridwell grade II at 26 levels (45%) in chambers with bioactive glass versus 33 levels (57%) in chambers with autologous bone, and Bridwell grade III at 2 levels (3%) in chambers with bioactive glass versus 2 levels (3%) in chambers with autologous bone (Table 2, Figs. 1 and 2). The 4 chambers that had fusion of Bridwell grade III (graft intact, but a definite lucency at the top or bottom of the graft) were observed in the L4-L5 cages of 2 patients that had undergone two-level stand-alone ALIF. The first was a 38-year-old woman, non-smoker, that had Bridwell grade I fusion at the L5-S1 chamber filled with bioactive glass, but grade II fusion at the L5-S1 chamber filled with autologous bone; she was reoperated 10 months after the index ALIF procedure, using posterior instrumentation filled with autologous local bone and allograft. The second was a 44-year-old woman, also non-smoker, that had Bridwell grade II fusion at both L5-S1 chambers; she was reoperated 23 months after the index ALIF procedure, also using posterior instrumentation filled with autologous local bone and allograft. Both patients that required reoperations were excluded from clinical assessment. There were no cases of cage subsidence, cage displacement, metal-plate migration, metal-plate fracture or bony fracture. For chambers filled with bioactive glass, there were no statistically significant differences in fusion rates among patients with posterior instrumentation versus those without at either L5-S1 (*p* = 0.755) or L4-L5 (*p* = 0.120). For chambers filled with autologous bone, there were no statistically significant differences in fusion rates among patients with posterior instrumentation versus those without at L5-S1 (*p* = 0.399), but fusion at L4-L5 was significantly better for patients with posterior instrumentation (*p* = 0.007).

**Table 2:** Fusion measured on computed-tomography scans using the Bridwell grade

	**Bioactive glass**				**Autologous bone**				
	Initial cohort	No posterior instrumentation	Posterior instrumentation		Initial cohort	No posterior instrumentation	Posterior instrumentation		
	n (%)	n (%)	n (%)	p-value*	n (%)	n (%)	n (%)	p-value*	p-value**
**Bridwell grade at any level (*n* = 58)**				*0.120*				*0.060*	*0.416*
I	30 (52%)	11 (19%)	19 (33%)		23 (40%)	11 (19%)	12 (21%)		
II	26 (45%)	8 (14%)	18 (31%)		33 (57%)	8 (14%)	25 (43%)		
III	2 (3%)	2 (3%)			2 (3%)	2 (3%)			
IV									
**Bridwell grade at L5-S1 (*n* = 40)**				*0.755*				*0.339*	*0.262*
I	21 (53%)	9 (23%)	12 (30%)		16 (40%)	8 (20%)	8 (20%)		
II	19 (48%)	7 (18%)	12 (30%)		24 (60%)	8 (20%)	16 (40%)		
III									
IV									
**Bridwell grade at L4-L5 (*n* = 18)**				*0.120*				*0.007*	*0.779*
I	9 (50%)	2 (11%)	7 (39%)		7 (39%)	3 (17%)	4 (22%)		
II	7 (39%)	1 (6%)	6 (33%)		9 (50%)		9 (50%)		
III	2 (11%)	2 (11%)			2 (11%)	2 (11%)			
IV									

*Abbreviations:* *SD* Standard Deviation, *n* Number of levels fused

* Comparison of patients with and without posterior instrumentation

** Comparison of chambers filled with bioactive glass and autologous bone

At a mean follow-up of 25 ± 2 months (range, 23–34), clinical assessment of the 38 remaining patients (54 levels) revealed that ODI improved from 48 ± 11 preoperatively to 15 ± 12 postoperatively (Table 3). Furthermore, lower back pVAS improved from 4.9 ± 1.4 to 1.4 ± 1.5 and legs pVAS improved from 3.7 ± 2.0 to 1.9 ± 1.6. Finally, the SF-12 physical component improved from 28 ± 6 to 45 ± 9 and the SF-12 mental component improved from 36 ± 8 to 46 ± 9. There were no statistically significant differences in postoperative clinical outcomes nor in the net change in clinical outcomes among the 24 patients with posterior instrumentation versus the 14 patients without.

**Table 3:** Pre- and post-operative clinical assessment

	**Final cohort (*n* = 38)**		**No posterior instrumentation (*n* = 14)**		**Posterior instrumentation (*n* = 14)**		p-value*
	mean ± *SD*	(range)	mean ± *SD*	(range)	mean ± *SD*	(range)	
**Follow-up (months)**	24.7 ± 2.4	(23 – 34)	25.4 ± 3.3	(23 – 34)	24.3 ± 1.6	(23 – 30)	*0.143*
**Lower back pVAS**							
*Preoperative*	4.9 ± 1.4	(2 – 8)	5.0 ± 1.2	(3 – 7)	4.9 ± 1.5	(2 – 8)	
*Postoperative*	1.4 ± 1.5	(0 – 6)	1.6 ± 1.8	(0 – 6)	1.3 ± 1.2	(0 – 4)	*0.742*
*Net change*	-3.5 ± 1.9	(-7 – 0)	-3.4 ± 2.0	(-7 – 0)	-3.6 ± 1.9	(-7 – 0)	*0.735*
**Leg pVAS**							
*Preoperative*	3.7 ± 2.0	(0 – 8)	3.5 ± 2.2	(0 – 7)	3.8 ± 2.0	(0 – 8)	
*Postoperative*	1.9 ± 1.6	(0 – 6)	2.4 ± 1.5	(1 – 6)	1.6 ± 1.6	(0 – 5)	*0.137*
*Net change*	-1.8 ± 2.8	(-8 – 5)	-1.1 ± 2.9	(-6 – 5)	-2.3 ± 2.7	(-8 – 2)	*0.207*
**ODI**							
*Preoperative*	47.9 ± 11.4	(32 – 72)	49.6 ± 12.1	(35 – 72)	46.9 ± 11.1	(32 – 72)	
*Postoperative*	14.8 ± 12.4	(0 – 54)	16.1 ± 14.0	(0 – 54)	14.0 ± 11.6	(0 – 42)	*0.647*
*Net change*	-33.1 ± 15.7	(-64 – 6)	-33.5 ± 16.7	(-64 -– 4)	-32.9 ± 15.4	(-62 – 6)	*0.910*
**SF-12 physical**							
*Preoperative*	27.5 ± 6.4	(16 – 44)	27.2 ± 7.0	(16 – 43)	27.7 ± 6.2	(17 – 44)	
*Postoperative*	45.4 ± 9.1	(20 – 59)	43.6 ± 9.7	(20 – 55)	46.5 ± 8.8	(24 – 59)	*0.340*
*Net change*	17.9 ± 9.4	(-9 – 36)	16.4 ± 9.0	(-1 – 32)	18.8 ± 9.7	(-9 – 36)	*0.214*
**SF-12 mental**							
*Preoperative*	35.8 ± 8.0	(22 – 53)	33.7 ± 8.0	(25 – 53)	37.0 ± 7.9	(22 – 50)	
*Postoperative*	46.4 ± 9.3	(21 – 59)	46.3 ± 11.2	(21 – 58)	46.5 ± 8.2	(27 – 59)	*0.705*
*Net change*	10.6 ± 13.2	(-32 – 37)	12.5 ± 15.9	(-32 – 28)	9.5 ± 11.6	(-8 – 37)	*0.203*

*Abbreviations: SD* Standard Deviation, *pVAS* pain on Visual Analogue Scale, *ODI* Oswestry Disability Index, *SF-12* Short-form 12

^*^ Comparison of patients with and without posterior instrumentation

## Discussion

The most important finding of this study is that, for ALIF at L5-S1 or L4-L5, fusion rates were equivalent for bioactive glass compared to autologous iliac crest bone, within the same patient. As reported for other ALIF implants [17, 19, 26], the present study found significant improvements of clinical outcomes at a follow-up ≥ 2 years, including ODI, lower back pain and leg pain. Therefore, the findings of this study suggest that for patients undergoing ALIF, bioactive glass can be used as a substitute to autologous iliac crest bone; thus, avoiding increased operative time and blood loss, as well as donor site morbidity [14, 22, 25]. While the follow-up of two years may not be sufficient to ascertain long-term clinical outcomes, the fusion rates of chambers filled with bioactive glass were already equivalent or better than the fusion rates of chambers filled with autologous bone graft, which led the authors to hesitate regarding the acquisition of further CT scans at longer follow-up, due to both ethical (exposure to radiation) and logistical (travel to radiology centers during the pandemic) considerations.

Comparing Bridwell grades observed in the present study suggests that fusion was better in chambers filled with bioactive glass (grade I in 52%) than in those filled with autologous bone (grade I in 40%), though the difference was not statistically significant (*p* = 0.416). There are two possible explanations for this trend: the first is that bioactive glass may induce better or faster bone growth; the second is that bioactive glass may appear more consolidated because it has greater radiopacity (Fig. 2). Considering Bridwell grades I and II to be satisfactory, the present study suggests fusion rates of 97%, both for bioactive glass and for autologous bone. These findings are similar to the only other published study that assessed ALIF using bioactive glass (combined with autologous bone), which reported a fusion rate of 100% at 1 year follow-up, in patients with neuro-compressive disorders at one to three lumbar levels [27]. Previous published studies on posterior fusion have reported fusion rates of 0–100% for bioactive glass (with or without autologous bone) [2–4, 11, 13, 15, 16, 22, 27], with only one of nine studies not recommending the use of bioactive glass [2] (Table 4). Furthermore, our fusion rate of 97% and complication rate of 5% are consistent with those reported for other studies investigating ALIF [5, 20, 26]. Of the 40 patients included in the present study, there were 2 patients that had to be reoperated because of inadequate fusion at L4-L5. It is important to note that both patients had undergone two-level stand-alone ALIF, and neither had posterior instrumentation. These findings suggest that when performing ALIF at two levels, posterior fixation may be necessary to stabilize the spine.

**Table 4:** Previous clinical studies reporting on the use of bioactive glass during spinal surgery

First author	Year	Type of surgery	Indication	Name of bioactive glass	Combined w/ bone	Comparator	Levels	n	Follow-up	Fusion rate of bioglass	Fusion rate of comparator	Recommend Bioglass
Westerlund [27]	2020	ACDF	Neurocompressive disorders	Bioactive glass bone graft (BioSphere Putty)	Yes, cancellous allograft		1–4 (cervical)	115	> 1 year	100%		Yes
		TLIF	Neurocompressive disorders	Bioactive glass bone graft (BioSphere Putty)	Yes, cancellous allograft		1–3 (lumbar)	30	> 1 year	100%		
		ALIF	Neurocompressive disorders	Bioactive glass bone graft (BioSphere Putty)	Yes, autologous bone		1–3 (lumbar)	103	> 1 year	100%		
Barrey [4]	2019	Posterior fusion	Degenerative diseases, trauma or spinal deformities	45S5 bioactive glass (GlassBoneTM, Noraker)	Yes (50:50)		2–10 (lumbar)	27	> 1 year	82%		Yes
		Posterior fusion	Degenerative diseases, trauma or spinal deformities	45S5 bioactive glass (GlassBoneTM, Noraker)	Yes (50:50)		1–2 (cervical)	3	> 1 year	33%		
Rantakokko [22]	2012	Posterior fusion	Burst fractures	BAG-S54P4	Yes	Autologous iliac crest bone	1–2 (lumbar)	16	10 years	50%	100%	Yes
Frantzen [11]	2011	PLF	Degenerative spondylolisthesis	BAG-S53P4	No	Autologous bone	2–3 (lumbar)	17	11 years	71%	100%	Yes
Ameri [3]	2009	Posterior fusion	Adolescent Idiopathic scoliosis	Metal-derived bioactive glass (Novabone)	Yes, local bone	Autologous iliac crest bone and local bone	Average 10 (thoracoumbar)	40	> 2 years	90%	85%	Yes
Acharya [2]	2008	PLF	Spondylolisthesis or stenosis	Hydroxyapatite-bioactive glass ceramic composite (Chitra- HABg)	Yes, bone marrow	Autologous bone	1–3 (lumbar)	24	> 1 year	0%	73%	No
Kasai [16]	2003	PLF	Stenosis	2:1 of bone:AWGC	Yes, autologous bone		2 (lunbar)	35	> 2 years	83%		Yes
			Stenosis	1:1 of bone:AWGC	Yes, autologous bone		2 (lunbar)	35	> 2 years	83%		
			Stenosis	1:2 of bone:AWGC	Yes, autologous bone		2 (lunbar)	35	> 2 years	82%		
Hashimoto [13]	2002	PLIF	Lumbar degenerative pathologies with instability	Bioactive ceramic granules (AWGC)	Yes, autologous bone		1 (lumbar)	25	> 2 years	100%		Yes
Ido [15]	2000	PLIF	Spondylolisthesis	AWGC	Yes, autologous bone		L4-L5	5	1.5 years 2 years	20% 50%		Yes
		PLF	Spondylolisthesis or vertebral fracture	AWGC	Yes, autologous bone		Multi (lumbar)	6	1.5 years 2 years	17% 50%		

*Abbreviations: AFPBP* Autogenous Fine Particulate Bone Powder, *BMSC* Bone Marrow mesenchymal Stem Cells, *ACDF* Anterior Cervical Decompression and Fusion, *TLIF* Transforaminal Lumbar Interbody Fusion, *ALIF* Anterior Lumbar Interbody Fusion, *PLF* Postero-Lateral Fusion, *RCT* Randomised Controlled Trial, *PLIF* Posterior Lumbar Interbody Fusion, *ICBG* Iliac Crest Bone Graft, *BMA* Bone Marrow Aspirate, *(TCP)* Tri-calcium Phosphate, *AWGC* Apatite-Wollastonite Glass–Ceramics, *n* number of patients

The present study has several limitations. First, comparisons between bioactive glass and autologous bone have been made within the same patient, and thus fusion or lack thereof in one chamber may have affected fusion in the other chamber; additionally, it is not possible to measure the effect of each material on postoperative clinical scores. Second, patients were operated on for a variety of indications, which may result in some variability in outcomes; although, this can also be regarded as a strength of the study since similar fusion rates were found for both materials across a range of indications. Third, ALIF cages of different sizes were used depending on the intervertebral height of each patient, which could mean that different cage sizes were filled with different amounts of material; however, this effect was diminished because we investigasted within-patient fusion rates, and the amount of filler material was equal for both chambers of each patient. Finally, the follow-up of the present study may not be sufficient to ascertain long-term clinical outcomes, although it is sufficient to evaluate fusion rates. Previous studies on other types of spinal surgery have demonstrated that early outcomes, such as ODI and Core Outcome Measures Index, improve or remain stable after 12 months and up to 8 years [1, 10].

## Conclusions

For ALIF at L5-S1 or L4-L5, within-patient fusion rates were equivalent for bioactive glass compared to autologous iliac crest bone. The findings of this study suggest that for patients undergoing ALIF, bioactive glass can be used as a substitute to autologous iliac crest bone; thus, avoiding increased operative time and blood loss, as well as donor site morbidity.

## Data Availability

The datasets used and/or analysed during the current study are available from the corresponding author on reasonable request.
